# The role of previous experience in the analysis of the psychological contract and its outcomes during the socialization process: a signalling theory perspective

**DOI:** 10.1080/09585192.2024.2401582

**Published:** 2024-09-16

**Authors:** Chris Woodrow, Ceren Erdem, David E. Guest

**Affiliations:** a Henley Business School, University of Reading, Reading, United Kingdom; b University of West London, Brentford, United Kingdom; c King’s Business School, King’s College London, London, United Kingdom

**Keywords:** Signalling theory, organizational socialization, psychological contract, occupational experience

## Abstract

Psychological contract theory has largely neglected the role of previous experience. In this study, we examine how previous work experience influences outcomes of communication with organizational insiders during organizational socialization among healthcare staff. We develop a model based on signalling theory, within which information acquisition during socialization is associated with psychological contract fulfilment, which is in turn is related to better health, happiness, and social relationships. Moderated mediation analysis based on data collected at entry and three months later confirms indirect effects between three types of information acquisition and three employee outcomes *via* the mediating role of psychological contract fulfilment. Importantly, these indirect effects are present only for inexperienced newcomers. Our findings build on signalling theory and add to knowledge about how the psychological contract forms during early socialization. They also suggest that organizations should pay particular attention to inducting inexperienced newcomers.

## Introduction

In certain sectors of employment, the need to attract and retain staff is a constant challenge. An important influence on the retention of newly recruited staff is the organizational socialization process, defined as ‘the process through which a new organizational employee adapts from an outsider to integrated and effective insider’ (Cooper-Thomas & Anderson, 2006, p. 492). A challenge for organizations is to provide relevant information and guidance to facilitate socialization. This is likely to be provided *via* a variety of sources including those involved in the recruitment process, those responsible for induction and any initial training, supervisors and, potentially, the human resource function (e.g. Guest et al., 2021; Kim & Moon, 2021; Walker et al., 2013).

The search for information and the receptiveness to and perceived value of this information among new recruits can vary. For example, there is likely to be a difference between newly recruited nurses who have done similar work in another hospital and nurses who have just graduated and are starting their first job. Those with previous relevant experience will come with pre-determined schema reflecting that experience and shaping what they look for, pay attention to, and value in the new organization (Sherman & Morley, 2015). Those without previous experience are likely to be hungrier for information and will utilise and value a variety of sources of information. However, we know little about what sources and types of information are valued by newcomers with more or less previous experience. Indeed, in understanding the search for and perceived usefulness of information and its impact of the socialization process, the potentially important role of previous experience has been largely neglected. There is therefore a need to explore the use and usefulness of information acquired during socialization, and its impact among those with more or less relevant previous experience. We focus upon three important types of information acquisition, namely that which occurs *via* interaction with insiders prior to entry, and *via* interaction supervisors and other organizational efforts after entry.

One important criterion in determining the effectiveness of socialization is the establishment of a psychological contract (Delobbe et al., 2016). The experiences and information gained during the early months of socialization play an important part in the development and then the fulfilment or breach of the psychological contract, particularly as it relates to promises made during the recruitment and initial induction periods. Those with previous experience and relatively established views about what to expect are likely to be more aware of promises that are more or less likely to be made and to be fulfilled (Tomprou & Nikolaou, 2011). While the content of the promises is important when considering the effectiveness of the socialization process, it is fulfilment of the promises that is likely to be more important in determining later employee well-being and retention. With a less clear understanding of what to expect, newcomers with no relevant experience are likely to be more uncertain about the nature and strength of promises and the credibility of the sources. They may therefore be more concerned about the non-fulfilment of promises than those with relevant experience who know what to expect and ‘have seen it all before’. There is therefore a need to study the difference in psychological contract fulfilment and its consequences among those with and without relevant previous experience.

There is an extensive body of research on psychological contract breach and its consequences for levels for organizational commitment, job satisfaction and intention to quit (Zhao et al., 2007). Much less research has explored fulfilment of the psychological contract and its implications for employee well-being. In the context of healthcare, which is the setting for our research, levels of well-being are likely to affect retention and also quality of patient care. We expect those with previous experience will be accustomed to the challenges of healthcare work. In contrast, those without relevant previous experience may be more likely to experience surprises and shocks that can affect their well-being. These may be largely ameliorated by an effective socialization process. We assess well-being *via* three components: psychological health, job satisfaction (as an indicator of happiness), and job embeddedness (as a measure of social relations).

In sum, there is a need to explore the relationship between the experience of socialization, the fulfilment of the psychological contract and the consequences for employee well-being, comparing those with and without relevant previous experience. To address these issues, and in particular the sources of information and their usefulness in aiding socialization and the fulfilment of the newly formed psychological contract, we use the lens of signalling theory.

Signalling theory (for a review see Connelly et al., 2011) has been widely used within various branches of management research (Tan, 2016). Its central concern has been to address information asymmetry and its consequences. One important context of such asymmetry arises when individuals join a new organization, and some of the early influential work on signalling theory addressed ways in which selectors of new recruits use information about education as a signal of potential employee quality (Karasek & Bryant, 2012). Research has also explored how job applicants gain necessary information about an organization from a variety of signals that the organization provides (Ehrhart & Ziegert, 2005; Rynes et al., 1991). We extend this approach, adopting a signalling theory framework to explore the sources used by new recruits to obtain information in the periods prior to and soon after joining a new organization. Specifically, we examine three sources of information that the socialization literature shows are important for newcomer integration: information obtained during recruitment, *via* supervisors, and *via* formal organisational material. We explore their impact on the psychological contract and related outcomes.

Despite being the focus of some studies of communication in the HRM field, signalling theory has paid limited attention to differences among receivers of signals, and how they affect outcomes of signalling (Drover et al., 2018; Tan, 2016). Yet, the motivation to search out relevant sources of information and evaluate the signals received is likely to vary among newcomers. One potentially important but largely neglected factor is previous work experience. Those with experience of the same type of organization and role—for example, nurses or physiotherapists in the healthcare field—may believe that they already have sufficient information, and therefore have little motivation to attend closely to signals from the organization. They will already have formed views about what to expect and about which sources and types of information are credible. This experience is also likely to influence their views about their psychological contract with the new organization. We will therefore explore the signalling process for those with and without relevant previous experience.

Signalling theory has not been applied to the study of how different sources of information affect individual outcomes, and in particular aspects of employee well-being among organization newcomers. We therefore extend signalling theory by applying it to an assessment of the sources of information that influence fulfilment of the psychological contract and the consequences for newcomer satisfaction, well-being, and job embeddedness, which are important outcomes for both individuals and organizations. We draw on signalling and psychological contract theory to develop and test a set of propositions about the sources of signals, the perceived usefulness of signals, and their implications for psychological contract fulfilment and associated outcomes among organizational newcomers who have different types of previous experience.

Our study makes several key contributions. First, it demonstrates the importance of signalling in the organizational socialization process and provides an extension of signalling theory to situations where both signallers and receivers are inside the organization. Second, it shows that psychological contract fulfilment is a key intervening mechanism in the link between information acquisition and employee outcomes, and highlights the important role of both pre-entry experience and post-entry knowledge acquisition in this process. Third, it highlights the importance of taking newcomers’ previous experience into account this context. In practical terms, our study strongly suggests that organizations should focus on identifying inexperienced ‘at risk’ newcomers and targeting them with communication, and highlights the important role of pre-entry signalling.

## Theory development

### Signalling theory

Signalling theory explores the nature and consequences of information asymmetry (Bergh et al., 2014; Connelly et al., 2011). It focuses on the signaller, the message, and the receiver, as well as considering the context and, potentially, the medium of communication. The signaller is usually an organization or its agents, while the receivers are anyone who might be influenced by a given message. The effectiveness of the communications depends on the qualities of the signaller and the signal as well as the willingness of the receiver to consider the message and view the source as credible (Gomulya & Mishina, 2017). In each case there is scope for considerable variability, and it is this that provides much of the research focus in signalling theory. In the present context, we focus on variability among receivers, and specifically on differences in the prior experience of newcomers.

Much research utilising signalling theory falls within the field of strategic management and considers a context of uncertainty about whether to support an investment opportunity. Potential investors will often search beyond the formally provided information for signals such as, for example, board membership, to determine whether the company looks like an investment-worthy proposition (Bergh et al., 2014; Connelly et al., 2011). There are direct parallels with the recruitment process in organizations. Recruiters send signals to potential recruits in the labour market designed to attract applicants of the right quality (Ehrhart & Ziegert, 2005). The temptation can be to place a positive gloss on the information provided, and receivers must determine the credibility of the source of information and its content. As well as behaviour in the labour market, organizations can send indirect signals. For example, being identified as one of the ‘100 Best Companies to Work For’ increased the number of high-quality applicants (Dineen & Allen, 2016). Receivers will also identify unintended signals. Rynes et al. (1991) found that applicants were influenced by factors including interview timing, delays in responding and perceived competence of the interviewers. Those with different levels of relevant previous experience are likely to interpret the signals in the recruitment process and in early socialization differently. Signalling theory can therefore extend our understanding of recruitment and socialization processes by focusing on the sources of information and their perceived usefulness for newcomers with different previous experience.

### The psychological contract

Most previous research using signalling theory has been concerned with organizational insiders sending signals and outsiders receiving them. We extend signalling theory to address signallers and receivers inside the organization by exploring its contribution to the analysis of the psychological contract. The psychological contract has been defined by Rousseau (1995:9) as ‘individual beliefs, shaped by the organization, regarding the terms of an exchange agreement between the individual and their organization’. It is distinct from the formal employment contract in that it refers to the employer and employee’s understanding of the employment relationship and their commitment to it (CIPD., 2023). In this study, we focus on the beliefs held by employees about mutual promises and obligations made in the context of their employment. Previous studies show that newcomers’ psychological contract beliefs change over time during early tenure (e.g. Robinson & Rousseau, 1994; Saks & Gruman, 2011). Changes can be very intense during the first months of tenure (e.g. Holtom et al., 2017; Thomas & Anderson, 1998), although they can continue much later than this (e.g. Rousseau et al., 2018). Early changes are influenced by information about the new organization, including the behaviour of individuals and organizational agents (e.g. Lee et al., 2011), and can predict employee attitudes and behaviour (e.g. Griep & Vantilborgh, 2018; Tekleab et al., 2013). However, there has been limited examination of the extent to which newcomer psychological contracts are affected by experiences prior to entry, or how this experience affects the impact of organizational signals. Previous scholars have alluded to the importance of signalling for the development and impact of the psychological contract (Rousseau, 1995; Suazo et al., 2009). Our analysis uses signalling theory to develop new insights into the formation of psychological contracts around the time of organizational entry and their consequences. In doing so, we incorporate insights from schema and sense-making theories.

There is a wide variety of potential signallers who can send signals about the content of the psychological contract and help to determine whether promises are kept. We focus on three important sources of signals commonly received by newcomers during socialization. The first is the recruitment and selection process, encompassing individuals with whom newcomers come into contact prior to entering the organization and associated recruitment material (e.g. Robinson & Morrison, 2000; Walker et al., 2013). The second is organizational induction, training and other official organizational messaging that occurs post-entry, which is likely to focus on the content of promises (e.g. Kim & Moon, 2021). The third is line managers and immediate supervisors who provide a primary point of contact and source of information for newcomers and will have a larger role in promise fulfilment (e.g. Pant & Venkateswaran, 2019). Newcomers are likely to be particularly attentive to signals that come from these sources, since credibility is likely to be signalled by positions of authority or expertise (Guest & Conway, 2002). The signals can take the form of explicit promises, but they are also likely to include inferred signals reflected in human resource policy and practice (Guest et al., 2021; Suazo et al., 2009), in mission statements (Fiset & Al Hajj, 2022; Turnley & Feldman, 2000) and in comments from different sources (Kammeyer-Mueller et al., 2013).

### Signalling and psychological contract development

In order to understand the way in which the psychological contract develops, we turn to schema theory. The early psychological contract and the associated beliefs held by newcomers can be understood as a developing schema. A schema refers to a mental model of conceptually related elements that are used to guide the way in which new information is organized (Coogan et al., 2022; Stein, 1992). Schema theory is an important approach in understanding how newcomers assimilate into organizations, as they are exposed to a lot of information that they must quickly organize and retain, Rousseau et al. (2018) stress that employees enter the organization with ‘normative expectations’ about the experiences and resources that they should receive, based on their previous experiences and pre-existing beliefs about employment relationships. This ‘anticipatory psychological contract’ acts as an initial frame of reference, or schema, which guides initial behaviour and beliefs, helping newcomers begin to make sense of their new environment (Tomprou & Nikolaou, 2011).

Psychological contract schemas will be modified during the ‘anticipation’ and ‘encounter’ stages of organizational socialization by signals from the organization through a process of sensemaking, which refers to the cognitive processes that individuals employ to cope with surprise and ambiguity (Maitlis & Christianson, 2014). Sensemaking helps individuals to measure how close their expectations are to reality (Weick, 1995) and can therefore precipitate perceptions of psychological contract fulfilment or breach. Sensemaking starts prior to organizational entry, during anticipation, when future employees start forming expectations of organizational life (Rynes et al., 1991) based on signals from the organization. At entry and early encounter, newcomers receive many signals about expected behaviour, experience various events, including ‘surprises’ (Louis, 1980) or even ‘shocks’ (Holtom et al., 2017), and actively seek signals to help them make sense of their new employment relationship. These signals trigger individuals to evaluate and adjust their existing expectations and beliefs (De Vos & Freese, 2011).

The content of newcomers’ psychological contracts is likely to be based partly on information provided during socialization and partly inferred from signals provided by the behaviour of other members of the organization. Prior to entry, the degree of pre-entry interaction (i.e. the level and frequency of communication with insiders) is a key indicator of later psychological contract fulfilment, because greater interaction enables newcomers to make more accurate sense of their surroundings (Robinson & Morrison, 2000; Walker et al., 2013), reducing misunderstandings in the deal. After entry, we focus on how useful the information received is deemed to be by the newcomers. Bergh et al. (2014) argue that individuals will seek out the most credible sources of information. These may be based on observable signals such as seniority, for example, line managers, or relevant specialist knowledge, such as human resource managers. As time goes by, they will come to rely on other sources of signals, such as supervisors with whom they have day-to-day contact. In addition, informal interaction creates scope for different signals from a variety of sources. There is a far greater array of potential sources of information post entry compared to pre-entry, and newcomers who are best able to identify signallers and signals they find more useful will be better able to make sense of their environment (Kammeyer-Mueller et al., 2013; Reichers, 1987). Hence, we propose that the perception of how useful information is will be the key to psychological contract fulfilment at this time. Useful signals will reduce ‘incongruence’ in the psychological contract, enabling sensemaking, helping to adjust schema to reality, and limiting the potential for post-entry misunderstandings between employer and newcomers about what has been promised and is being delivered. We expect psychological contract fulfilment to represent what Bergh et al. (2014) refer to as ‘signal confirmation’ in newcomers. We therefore offer the following hypothesis:

**H1:** Socialization information [pre-entry interaction (1a); supervisor information usefulness (1b); organizational information usefulness (1c)] is positively associated with psychological contract fulfilment.

#### Previous experience and psychological contract development

Psychological contract theory has identified information gained during ‘occupational socialization’ and prior to contact with the current employer as an important basis for the development of the psychological contract (Rousseau et al., 2018). Studies to date have often examined the issue of newcomer psychological contracts by focussing only upon neophyte newcomers (i.e. those new to both their occupation and their organization) using samples of graduates entering the workforce (Lee et al., 2011) or new recruits to organisations such as the army (e.g. Thomas & Anderson, 1998). Other studies have examined newcomer psychological contracts using samples of newcomers with varying levels of previous experience that are treated as one homogenous group (e.g. De Vos & Freese, 2011). A gap in our knowledge therefore remains about the role of relevant previous experience in psychological contract development and its outcomes. We address this issue by exploring how the perceived usefulness of signals that newcomers receive affects early psychological contract fulfilment in those with and without recent relevant previous experience. We focus on the most recent role held by newcomers as being of particular influence in this process, because recency effects operate with respect to schema development (e.g. Peasley et al., 2021; Sherman & Morley, 2015; Srull & Wyer, 1980), such that more recently used schemas will be more easily accessed, retrieved, and enacted in future situations.

The receivers of the signals, the employees and more particularly newcomers whose psychological contracts are developing, will differ in the way they receive, interpret and internalise signals about the content and subsequent fulfilment of the psychological contract. Signalling theory has incorporated screening theory (Sanders & Boivie, 2004) which addresses the way in which receivers pay attention to the signaller and signals that they find most credible and useful. For example, Sauzo et al. (2009) have suggested that HR practices provide potentially important signals about implicit organizational promises. Ostroff and Bowen (2016, p. 197) note that ‘HR practices send signals to employees about the responses and behaviors that are expected rewarded and valued. These signals are interpreted by employees’. Guest and Conway (2002) found that the presence of more HR practices was perceived by managers as the form of signalling most strongly associated with an explicit psychological contract.

During early socialization, inexperienced newcomers are ‘information needy’, displaying ‘signal susceptibility’ or a readiness to believe that signals are credible. Because there will be both explicit and implicit signals, there is likely to be a lot of ‘noise’. When coupled with prior expectations, this can make it difficult, especially for newcomers, to distinguish signals that constitute promises from those reflecting less specific intentions. Drover et al. (2018) propose a dual process model of reaction to signals. They suggest that individuals will initially use ‘heuristics’ based on established decision rules that draw on previous experience. If this is insufficient or individuals lack relevant experience, they will engage in a more effortful conscious information search and processing of the received signals. Building on Drover et al’s analysis, we argue that those with relevant previous experience are more likely to use heuristics to make sense of their new environment, while other newcomers without established schema are more likely to engage in conscious information processing.

Schema theory can help us to understand how these processes affect the development of perceptions of breach or fulfilment. Inexperienced newcomers will be uncertain in their new environment and are therefore particularly attentive to signals about the developing employment relationship (Kammeyer-Mueller et al., 2013; Morrison & Robinson, 1997). They are more likely to search for signals, which will be deemed to be useful when they ‘flesh out’ the existing schema. This process enables them to learn about generally acceptable behaviour and decreases misunderstandings between employees and agents of the organization around what has been promised. Consequently, they are more likely to experience psychological contract fulfilment after receiving information that is deemed to be useful.

Conversely, those with experience of doing the same kind of work in the same kind of context know what to expect; in signalling terms, they have a form of ‘insider knowledge’. They hold psychological contracts that are more developed and harder to change in the face of new information compared to inexperienced colleagues (Rousseau, 2001; Tomprou & Nikolaou, 2011). Signals will be perceived as useful if they confirm existing schema, reinforcing rather than changing their psychological contract and expectations of fulfilment. They may already have set views about the content and likely fulfilment of the psychological contract as well as the type of organization they have joined, and will fall back on their existing beliefs, using heuristics to assess the signals and their signallers. Useful information is therefore unlikely to influence the extent to which they perceive psychological contract fulfilment. Based on this analysis, we make the following hypothesis:

**H2:** The relationships between different types of socialization information [pre-entry interaction (2a); supervisor information usefulness (2b); organizational information usefulness (2c)] and psychological contract fulfilment are moderated by previous relevant experience, such they are weaker among those with relevant experience.

### Previous experience, the psychological contract and outcomes

We expect relevant pre-entry experience to affect reactions to signals from the organization to newcomers, and subsequently their psychological contracts. There is evidence that various pre-entry knowledge of organizational life is associated with proximal and distal post-entry indicators of adjustment (Kammeyer-Mueller & Wanberg, 2003). These outcomes serve, in effect, as indicators of signalling effectiveness. One of the important indicators of both proximal and distal adjustment is an individual’s work-related well-being, which is an outcome that has been largely neglected in previous research on the psychological contract despite its central relevance to employees (for exceptions, see Conway et al., 2011; Zacher & Rudolph, 2021). We therefore focus on well-being, and follow the approach of Grant et al. (2007) in viewing well-being as comprising three components covering health, happiness and social relations. We use a standard measure of anxiety-contentment to explore psychological health, job satisfaction as an indicator of happiness, and job embeddedness as a measure of social relations. The concept of job embeddedness (Mitchell et al., 2001) addresses the strength of links to people and activities at work, the fit between current work and other aspects of life and the social and other sacrifices associated with leaving current work (Lee et al., 2014). Although often studied in relation to later tenure, there is strong evidence that job embeddedness is fostered during early socialization (e.g. Allen, 2006; Peltokorpi et al., 2022; Rubenstein et al., 2019) and that it has been shaped by the three-month time point used in the current study (Allen & Shanock, 2013) through the extensive interactions that occur during this period. We propose that those reporting higher levels of fulfilment of the psychological contract will be more likely to report positive outcomes, since the perceived delivery of promises signals goal achievement and creates the perception that one is valued at work, leading to an emotional uplift and increased trust (e.g. Guerrero & Herrbach, 2008; Robinson & Rousseau, 1994).

**Figure 1.**
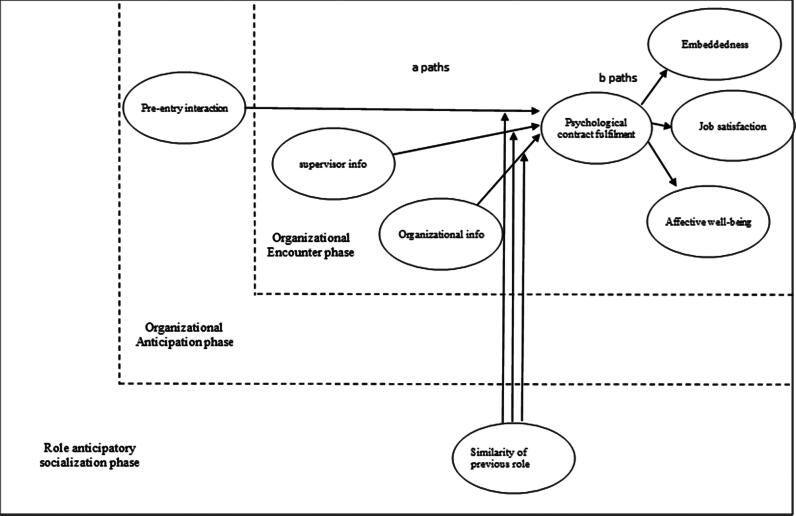
A model of signalling and the psychological contract during socialization.

We propose that an indirect effect between information usefulness and these outcomes *via* psychological contract fulfilment will be moderated by relevant previous experience, on the grounds that those with previous experience have been less influenced by the signals from the organization and relied more on their established schema. These schemas will have helped to provide a realistic view about the likely levels of psychological contract fulfilment, meaning that they are more likely to avoid any shocks or surprises (Holtom et al., 2017; Louis, 1980) resulting from non-fulfilment. As a result, they have more realistic expectations and are less surprised by non-fulfilment of their psychological contract. In contrast, and in line with an extensive body of previous research, those without the relevant previous experience and associated schema will react to failure by the organization to fulfil the psychological contract with disappointment and potential damage to their health, happiness and social relationships. Figure 1 shows all study hypotheses.

**H3:** Psychological contract fulfilment will be positively associated with affective well-being [3a], job satisfaction [3b] and embeddedness [3c].

**H4:** The indirect effect of socialization information (pre-entry interaction; supervisor information usefulness; organizational information usefulness) on employee outcomes (affective well-being; job satisfaction; embeddedness) via psychological contract fulfilment will be moderated by previous relevant experience, such the relationships are weaker among those with relevant experience.

## Methodology

### Participants and procedure

Participants joining a large general hospital in the UK were recruited into the study with a questionnaire that was administered during the first month in their new role at an organizational induction session (T1). Participants were provided with an information sheet and provided consent to participate *via* completion of this initial survey. At this stage, participants provided contact details for follow up. The follow up survey was sent *via* both email and post at three months (T2) of service, which is considered to be close to the end of the ‘encounter’ stage of socialization (Bauer et al., 2007). Of 345 Individuals working in the healthcare sector who participated at T1 and provided consent for follow-up, 162 responded at T2. Participants were a mix of individuals from across various healthcare professions, though the emphasis in recruitment was on occupations such as nursing resulting in a bias in the sample towards female newcomers. Table 1 shows demographic information for the final sample.

**Table 1.** Sample demographics.

Characteristic	Statistics
Age	
Range	20-59
Mean	33
Occupation	
Nurse	79 (48.8%)
Other clinical	34 (21.0%)
Administrative	43 (26.5%)
Gender	
Male	23 (14.2%)
Female	139 (85.8%)

### Measures

Table 2 shows relevant alpha coefficients, means, standard deviations and intercorrelations across study variables. ‘Previous Role’ was assessed at T1 using two items. The first was a forced choice item asking what activity participants were engaged in ‘directly before they joined’ the organization, with options for ‘unemployed’, ‘working’, ‘training/education’, and ‘other’. Participants were also invited to enter additional details in a free text field. These two items were used to compute a dichotomous variable to indicate whether individuals had been engaged in similar employment directly prior to entry.

**Table 2.** Study variables, descriptive statistics and intercorrelations.

Variable	Mean (SD)	α	1	2	3	4	5	6	7	8	9	10
1. Age	32.88 (8.83)	--	--									
2. Gender	--	--	0.01	--								
3. Similar employment	--	--	0.17*	0.03	--							
4. Pre-entry interaction	3.20 (0.93)	0.74	−0.11	0.01	−0.05	--						
5. Supervisor info usefulness	2.82 (0.96)	0.75	−0.01	−0.08	−0.02	0.17*	--					
6. Organizational info usefulness	2.87 (0.97)	0.80	−0.03	0.01	−0. 03	0.20*	0.32**	--				
7. PC fulfilment	4.12 (0.70)	--	0.01	0.15	0.12	0.15	0.32**	0.26**	--			
8. Job embeddedness	2.85 (0.78)	0.88	0.09	0.02	0.07	0.20*	0.24**	0.19*	0.24**	--		
9. Affective well-being	3.68 (0.73)	0.79	0.04	−0.02	0.01	0.09	0.32**	0.14	0.49**	0.33**	--	
10. Job satisfaction	4.27 (0.89)	0.94	0.05	0.07	0.09	0.12	0.29**	0.21**	0.58**	0.37**	0.70**	--

‘Pre-entry interaction’ was assessed at T1 using four items from Robinson and Morrison (2000) assessing level and frequency of communication with organizational insiders prior to day one in the job of (e.g. ‘during the recruitment process, I talked in depth with people at [the organization]’). Responses were on a five-point scale from strongly agree (5) to strongly disagree (1). ‘Supervisor information usefulness’ and ‘Organizational information usefulness’ were assessed at Time 1 *via* six items developed from Morrison (1993) and Kammeyer-Mueller and Wanberg (2003). These assessed the usefulness of information obtained from the two sources concerning three of the most important types of information obtained during early socialization (e.g. Haueter et al., 2003), namely information about the team, role, and the organization as a whole. To assess ‘Supervisor information usefulness’, three items assessed whether participants had consulted ‘their direct supervisor’ in order to determine a) how to perform specific aspect the job, b) the rules, goals and values of their new team, and c) the rules, goals and values of their organization as a whole. ‘Organizational information usefulness’ was assessed by repeating these three items with reference to information provided ‘during induction, training or other efforts made by the organization’. Respondents rated each item to indicate whether information had been obtained. If so, respondents were asked to indicate how useful this information had been, using a 5-point Likert scale (1 = No; 5 = Yes, and this was very useful)

‘Psychological Contract fulfilment’ was assessed at T2 *via* a composite score computed from a set of 18 potential promises and obligations. For each item, participants were asked to rate whether their employer had made such a promise, either informally or formally, and how far it had been delivered. Items were scored *via* a six-point Likert scale ranging from 0 (‘not at all’) through 1 (‘promise made but not delivered’) to 5 (Promise made and delivered to a great extent’). The fulfilment score was produced by computing the mean value of items where the respondent indicated that a promise had been made. The initial set of promises were based upon those developed by Guest et al. (2010) using factor analysis, pilot work, literature reviews, and expert discussion. We adapted these items to the study context *via* further pilot work that involved interviews with 18 individuals working at the host organization to elicit relevant promises and obligations. This process resulted in the adaptation of some of the terminology used to describe promises towards healthcare-specific terms. By the time we had undertaken 18 interviews no new issues were raised by interviewees. Example items include ‘Support you in providing the highest possible quality of service to patients/service users’ and ‘Be flexible in matching the demands outside of work with your job’. The full list of items is available from the corresponding author upon request.

‘Job Embeddedness’ was assessed at T2 *via* Crossley et al.’s (2007) six-item global embeddedness measure, (e.g. I am tightly connected to [the organization]. Job satisfaction was assessed using four items based on Cammann, Fichman, Jenkins, and Klesh’s, (1979) scale (e.g. ‘In general, I like working here’). Affective well-being was assessed using six items from Warr’s (1990) job-related affect scale (e.g. ‘calm’; ‘tense’), with participants indicating how far they had felt each well-being indicator at work in the past few weeks. All items making up these three constructs were assessed at T2 and rated on a five-point Likert scale ranging from strongly agree to strongly disagree.

### Analysis

Hypotheses were examined *via* moderated mediation regression analyses using the Process Macro for SPSS (version 3.5; Hayes, 2018). This involved running a series of ordinary least squares regression models to establish the relationships between our proposed predictors (pre-entry interaction; supervisor information usefulness; organizational information usefulness), mediator (psychological contract fulfilment), and outcome (job embeddedness; job satisfaction; affective well-being) variables, at different levels of the proposed moderator (similarity of previous employment), thereby testing all study hypotheses. Age and gender were entered into each model as control variables, since there is some evidence that each is associated with employee perceptions of psychological contract expectations and breach (e.g. Coyle-Shapiro et al., 2019; Ng & Feldman, 2009). Continuous predictor variables were centred prior to analysis.

The initial analysis examined the left side of our proposed model, including the predictors of psychological contract fulfilment (the ‘a’ paths) and the moderating effect of similar employment. Turning first to the role of pre-entry communication, psychological contract fulfilment was regressed on the pre-entry communication variable, enabling us to test hypothesis 1a. In order to assess hypothesis 2a, we included the similar employment variable and an interaction term (pre-entry experience x similar employment) as predictors in this model. We further probed the interactions by comparing and graphically plotting the relationship between pre-entry experience and psychological contract fulfilment among those joining from similar employment and those joining from elsewhere. We repeated this process with the supervisor information usefulness and organization information variables, allowing us to test hypotheses H1b, H1c, H2b and H2c.

The second part of the analysis examined the right side of our proposed model i.e. the relationship between fulfilment and the outcome variables (the ‘b’ path). This involved regressing each of the outcome variables on psychological contract fulfilment and pre-entry experience, allowing us to examine H3a-H3c. We examined H4, concerning moderation of the total indirect effect, in several steps. First, we calculated conditional indirect effects for each level of the moderator variable by computing the product of the relevant a and b paths. Indirect effects were then assessed using a bootstrapping technique based on 5000 samples. Analysis of indirect effects was based on the method outlined by Preacher and Hayes (2004), which does not require a direct predictor - outcome effect to be present in order to establish an effect. Second, we assessed the index of moderated mediation (Hayes, 2018) in order to establish whether the effect of the moderator on the total indirect effect was significantly different to zero.

## Findings

**Figure 2.**
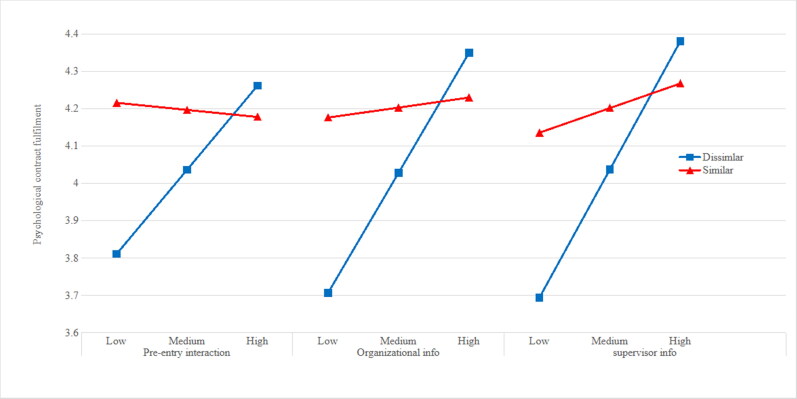
The relationship between information acquisition and PC fulfilment across similar v dissimilar previous employment types.

All findings from the moderated mediation analyses that we used to examine hypotheses 1- 4 are detailed in Tables 3–5. As shown in the ‘model one’ column of each table, pre-entry interaction, supervisor information and organizational information are each significantly positively related to psychological contract fulfilment. Hypotheses 1a, 1b and 1c are therefore supported. Additionally, the interaction terms in each model are significantly different from 0, indicating that each of the relationships are moderated by previous role. Figure 2 shows interaction plots for each of these relationships. In each case, there is a significant positive relationship between information acquisition and psychological contract fulfilment for those joining from dissimilar jobs or education. but not for those joining from similar jobs. Hypotheses 2a, 2b and 2c are therefore supported. The ‘model two’ columns of each table indicate that fulfilment of the psychological contract is significantly associated with each of the outcome variables (affective well-being, job satisfaction, and embeddedness) in each model. H3a, H3b and H3c are therefore supported.

**Table 3.** Moderated mediation results for pre-entry interaction analyses.

	Model 1	Model 2		
	PC fulfilment	Well-being	Embeddedness	Job satisfaction
Predictor				
Control variables^a^				
Age	0.00 (0.01)	−0.01 (0.01)	0.01 (0.01)	0.01 (0.01)
Gender	0.30 (0.16)	−0.02 (0.10)	−0.06 (0.18)	−0.06 (0.17)
Independent variables^a^				
Pre-entry interaction	0.24 (0.08)**	0.03 (0.05)	0.17 (0.07)*	0.03 (0.07)
PC fulfilment		0.49 (0.07)**	0.23 (0.09)**	0.74 (0.09)**
Similar employment	1.01 (0.41)*			
Interaction terms^a^				
Pre-entry interaction x Similar employment	−0.26 (0.12)*			
Model statistics				
R^2^	0.09	0.26	0.10	0.35
F	2.99*	12.86**	4.14*	20.01**
Conditional Indirect effects^b^				
Dissimilar employment		0.12 (0.03,0.22)	0.06 (0.01, 0.13)	0.19 (0.06, 0.34)
Similar employment		−0.01 (−0.09, 0.07)	0.06 (0.01, 0.13)	−0.01 (−0.12, 0.12)
Moderated mediation effects^b^				
Index of moderated mediation		−0.13 (−0.26, −0.02)	−0.06 (−0.015, −0.01)	−0.20 (−0.38, −0.03)

^a^Entries are standardised regression coefficients; bracketed values represent standard errors.

^b^Entries are effect sizes; bracketed values represent confidence intervals.

*p < 0.05; **p,0.01.

**Table 4.** Moderated mediation results for supervisor information analyses.

	Model 1^a^	Model 2^a^		
	PC fulfilment	Well-being	Embeddedness	Job satisfaction
Predictor				
Control variables				
Age	−0.02 (0.01)	0.01(0.01)	0.01(0.01)	0.00 (0.01)
Gender	.036 (0.15)*	−0.18(0.14)	−0.01(0.18)	−0.02 (0.17)
Independent variables				
Supervisor info	0.37 (0.07)**	0.15(0.05)**	0.14(0.07)*	0.13 (0.07)
PC fulfilment		0.14(0.07)**	0.20(0.09)*	0.69 (0.09)**
Similar employment	0.16 (0.11)			
Interaction terms				
Supervisor info x Similar employment	−0.30 (0.12)*			
Model statistics				
R^2^	0.18	0.29	0.09	0.36
F	6.60**	15.39**	3.68**	21.36**
Conditional Indirect effects^b^				
Dissimilar employment		0.16 (0.08, 0.26)	0.07 (0.01, 0.16)	0.25 (0.13,0.41)
Similar employment		0.03 (−0.05, 0.13)	0.01 (−0.02, 0.07)	0.06 (−0.08,0.19)
Moderated mediation effects^b^				
Index of moderated mediation		−0.13 (−0.26, −0.02)	−0.06 (−0.14, −0.01)	−0.20 (−0.40, −0.03)

^a^Entries are standardised regression coefficients; bracketed values represent standard errors.

^b^Entries are effect sizes; bracketed values represent confidence intervals.

*p < 0.05; **p,0.01.

**Table 5.** Moderated mediation results for organizational information analyses.

	Model 1^a^	Model 2^a^		
	PC fulfilment	Well-being	Embeddedness	Job satisfaction
Predictor				
Control variables				
Age	0.00 (0.01)	0.00 (0.01)	0.01 (0.01)	0.00 (0.01)
Gender	0.28 (0.16)	−0.23 (0.14)	−0.05 (0.18)	−0.06 (0.17)
Independent variables				
Org info	0.33 (0.08)**	0.01(0.05)	0.11 (0.07)	0.06 (0.06)
PC fulfilment		0.49 (0.07)**	0.22 (0.09)*	0.73 (0.09)**
Similar employment	0.17(0.11)*			
Interaction terms				
Org info x Similar employment	−0.30(0.11)*			
Model statistics				
R^2^	0.15	0.26	0.08	0.35
F	5.01**	12.77**	3.29*	20.22**
Conditional Indirect effectsb				
Dissimilar employment		0.16 (0.08,0.26)	0.07 (0.02,0.15)	0.24 (0.12,0.41)
Similar employment		0.01 (−0.06, 0.08)	0.01 (−0.03, 0.04)	0.03 (−0.08,0.13)
Moderated mediation effectsb				
Index of moderated mediation		−0.15 (−0.27, −0.05)	−0.07 (−0.15, −0.01)	−0.22 (−0.41, −0.06)

^a^Entries are standardised regression coefficients; bracketed values represent standard errors.

^b^Entries are effect sizes; bracketed values represent confidence intervals.

* *p* < 0.05.

** *p*,0.01.

Finally, the index of moderated mediation in each model is significantly different to 0, indicating that similar previous employment moderates the indirect effect of information acquisition on outcomes *via* psychological contract fulfilment. In order to explore this effect, we calculated the conditional indirect effects at each level of the moderator, as shown in Tables 3–5. In each case, there is a significant positive indirect effect between each socialization entry variable (*pre-entry interaction; supervisor information usefulness; organizational information usefulness)* and each outcome variable (*affective well-being; job satisfaction; embeddedness*) *via* psychological contract fulfilment. This indirect effect holds only for those coming from dissimilar roles; there is no such indirect effect for those coming from similar roles. Taken together, the findings indicate an indirect effect as well as moderated mediation, and provide strong support for H4.

## Discussion

We have used signalling theory to explore the value of different sources of information for the fulfilment of the psychological contract among organizational newcomers. In doing so, we have focussed on the receivers of these signals. As Drover et al. (2018) have noted, signalling theory has largely neglected individual differences in characteristics of those receiving the information. We have sought to remedy this deficiency by exploring the impact of differences in recent relevant experience in other organizations. Our study confirms that these differences influence the impact of signals from various organizational sources on fulfilment of the psychological contract. We followed Earnest et al. (2011) in focusing on signals received soon after entry into the new organization, confirming their importance; useful information at this point was associated with a more fulfilled psychological contract. These signals complemented information gained prior to entry, which also contributed to psychological contract fulfilment. Our findings provide important implications for our understanding of the organizational socialization process and the role of signalling within it.

At an overarching level, this study contributes to the literature by confirming the utility of signalling theory as an analytic framework for exploring the development and impact of the psychological contract. Our research makes several important contributions in this area. First, we extend signalling theory to situations where both signallers and receivers are within the organization, where the focus of much previous research has been on signals that are sent outside of the organizations. Second, we also extend Drover et al. (2018) dual process cognitive model of signalling to describe the way in which organizational newcomers process information in their new environment. Our study suggests that those with previous relevant experience are likely to use heuristics to make sense of their new environment, contributing to the development of the psychological contract, compared to newcomers without relevant experience. In sum, our study places emphasis on the sender or sources of information, the content or usefulness of the messages and the characteristics of the receivers, all of which provide insights into the development and fulfilment of the psychological contract and into employee well-being.

A further key contribution of our research is the finding that all sources of information, whether provided by the sender, the organization, or actively sought by the receiver, the newcomer, contribute to psychological contract fulfilment. Our data showed no evidence of significant differences between newcomers with relevant previous experience and other newcomers in the perceived usefulness of information sought or provided (based on correlational analysis), but it did reveal significant differences in the impact of this information. Specifically, as hypothesised, information had a weaker association with fulfilment of the psychological contract among those with relevant previous experience, such that previous experience moderated (and weakened) the indirect association between information acquisition and three indicators of psychological well-being, *via* psychological contract fulfilment. An important finding of our study is therefore the importance of both pre-entry experience and post-entry knowledge acquisition for effective socialization, which has been largely neglected in previous research.

Following the approach of Rousseau (1995, 2001) and Coogan et al. (2022), we interpret these findings through the lens of schema theory. Those with previous relevant experience hold established schema that help to shape expectations and are relatively resistant to change through new information. In signalling terms, Drover et al. (2018) explain this as the use of established heuristics. More experienced newcomers are less influenced by new information—and in the case of promises associated with the psychological contract, less likely to accept them as wholly credible - whereas newcomers without relevant experience and associated schema to fall back on will be more receptive to the information they receive as they develop new schema, and potentially more disturbed if they find that the promises about what to expect, reflected in the information obtained, are not kept. In short, those with relevant previous experience will have more realistic expectations about fulfilment of the psychological contract, while failure to keep promises has more negative consequences for less experienced newcomers. Our analysis therefore helps to explain how relevant individual differences can impact the socialization process, advancing our understanding of variations in socialization experience.

A final key contribution of our paper is the exploration of the link between psychological contract fulfilment and three measures of employee well-being. We followed the widely used approach to well-being that focuses on health, happiness and social relations (Grant et al., 2007). There has been extensive research exploring the association between the psychological contract and job satisfaction (Coyle-Shapiro et al., 2019; Zhao et al., 2007), but far less examining psychological well-being and social relations. We expected that at the end of the encounter stage of socialization, the content of the signals received by newcomers would affect their well-being *via* the mediating mechanism of psychological contract fulfilment. Our results confirm this for all three dimensions of well-being and show that this impact is weaker among those with relevant previous experience. These findings are particularly important in sectors like healthcare, where retention and staff morale are often a challenge.

### Practical implications

Our study raises practical implications that may assist managers and HR professionals to facilitate healthy employment relationships. First, this study highlights the importance of taking previous relevant experience into account in organizational selection and socialization, suggesting that newcomers with similar and dissimilar previous experience may require quite different support upon entry. For newcomers with dissimilar or very little experience, strong and clear signals about organizational contributions should be the priority around entry in order to help them settle into organizational life and promote better well-being. Our study shows that signals from both the organization system and line managers are important here.

Our study also highlights the importance of signalling to newcomers prior to entry. Whilst such communication may often be overlooked, our study shows its importance in promoting future psychological contract fulfilment and well-being, especially for newcomers without similar experience. Through job advertisements and effective signalling of values and realistic job previews, organizations can attract not only the most talented, but also the best fitting individuals. HR professionals should therefore create strategies to effectively communicate employee and employer obligations to potential recruits in their new role, such that both parties can understand each other’s expectations at the initial stage of the employment relationship. Once an offer is made, ongoing communication appears to continue to benefit the formation of healthier employment relationships and better well-being outcomes. In sum, our study suggests that organizations should actively engage in the communication of realistic information to newcomers prior to their entry.

### Limitations and future research

Despite the strengths in our study and its findings, as outlined above, there are some limitations in our study which point the way towards future research. The first concerns the sample. Study participants were all healthcare workers in the UK, and data collected in other contexts may lead to different findings. For example, the location of newcomers’ previous experience may also be an important factor in socialization, since healthcare systems differ quite substantially by country. Future studies may examine these findings more widely and in different contexts. Second, our study had a limited sample size, meaning we were not able to test all antecedents in one model. Future research could address some of these possible antecedents by using a larger sample. Third, we considered only the influence of the immediate previous role of study participants. However, we acknowledge that the nature of the immediate previous role might also have an influence on psychological contract formation. For example, whether the immediate past role is consistent with previous experience or a temporary career change might influence the way signals are received by newcomers. Future research might further explore how the nature of the immediate past role influences the proposed relationships.

Fourth, and related, the focus of this paper has been on a particular individual difference, concerning previous experience. Given the sample in the survey was skewed towards female healthcare workers, we controlled for age and gender. However, other individual differences may also potentially influence study findings. Personality and predispositions of employees may influence newcomers’ perceptions of the psychological contract and propensity to attend to signals (e.g. Kammeyer-Mueller & Wanberg, 2003). Motivation and qualifications could affect the way pre-entry signals are received, whilst post entry factors such as indicators of the fit between the supervisor and the new recruit, may influence sensemaking behaviors among newcomers and how far information is attended to. In sum, future research should further consider the various aspects of newcomers’ background, gender, identity, personality and post-entry experiences in the way that signals are received during socialization.

A fifth limitation concerns the sources of signals examined and their outcomes. We focussed on three key sources—recruitment, managers, and formal socialization—reasoning that these were likely to be amongst the most salient. However, there are other sources of signals that could be influential here. Indeed, the socialization literature has identified various sources of information, including co-workers, mentors, sponsors, and task-related feedback (e.g. Kim & Moon, 2021; Morrison, 1993) that can affect socialization. Moreover, there are several methods of acquiring such information (e.g. questioning; observing) several types of information content (e.g. technical; feedback-related), and different methods of delivering such information, such as in group vs. individual form (e.g. Jones, 1986; Saks and Gruman, 2011). There are also various proximal level indicators of adjustment, such as role clarity, and different types of psychological contracts (i.e. transactional or relational), which may be more or less affected by signals. In sum, future research may further explore the role of signalling across these various modalities.

Finally, this study focuses on the formation of psychological contract before entry and during the first three months of the employment. Indeed, the literature has for some time indicated that the psychological contract sees very large changes during the first three months (e.g. Ashforth, 2012; Thomas & Anderson, 1998). However, it is also acknowledged that psychological contract does continue to change after this, e.g. at up to two years of service (e.g. Robinson et al., 1994; Rousseau et al., 2018). Therefore, future research may further explore psychological contract formation and the way signals are received, as well as what signal sources matter most, beyond the initial three months of socialization.

## Data Availability

Data cannot be shared for this article due to privacy considerations.
